# Honeycomb, a New Food Resource with Health Care Functions: The Difference of Volatile Compounds found in *Apis cerana* and *A. mellifera* Honeycombs

**DOI:** 10.3390/foods11203204

**Published:** 2022-10-14

**Authors:** Xiali Guo, Yanlang Liang, Shengxiang Yi, Shengrong Qiu, Mingyan Liu, Fangjian Ning, Liping Luo

**Affiliations:** 1 School of Life Sciences, Nanchang University, Nanchang 330031, China; 2 School of Food and Health, Beijing Technology and Business University, Beijing 100048, China; 3 State Key Laboratory of Food Science and Technology, Nanchang University, Nanchang 330031, China

**Keywords:** honeycomb, *Apis cerana*, *A. mellifera*, HS-SPME/GC-MS, VOCs

## Abstract

The honeycomb composition is very complex, containing honey, royal jelly, pollen, and propolis, and thus contains a large number of bioactive ingredients, such as polyphenols and flavonoids. In recent years, honeycomb as a new functional food resource has been favored by many bee product companies, but the basic research on honeycomb is lacking. The aim of this study is to reveal the chemical differences between *A. cerana* honeycombs (ACC) and *A. mellifera* honeycombs (AMC). In this paper, we studied the volatile organic components (VOCs) of ACC and AMC by solid-phase microextraction gas chromatography-mass spectrometry (HS-SPME/GC-MS). A total of 114 VOCs were identified in 10 honeycombs. Furthermore, principal component analysis (PCA) revealed that the chemical composition of ACC and AMC were different. Additionally, orthogonal partial least squares discrimination analysis (OPLS-DA) revealed that benzaldehyde, octanal, limonene, ocimene, linalool, α-terpineol, and decanal are the significant VOCs in AMC extracts, which are mainly derived from propolis. OPLS-DA model also identified 2-phenylethanol, phenethyl acetate, isophorone, 4-oxoisophorone, betula, ethyl phenylacetate, ethyl palmitate, and dihydrooxophorone as potential discriminatory markers of ACC, which likely contribute to protecting the hive against microorganisms and keep it clean.

## 1. Introduction

Honeycomb is a waxy structure built by worker bees from 12 to 18 days old using abdominal wax gland secretions, where bees inhabit, breed, and store food [1,2]. Honeycomb is mixed with other bee products, such as honey, royal jelly, pollen, and propolis. Therefore, the honeycomb components are very complex, containing not only abundant beeswax components but also a large number of biologically active components, such as polyphenols and flavonoids [3,4]. Honeycomb has been recorded by Shen Nong’s *Herbal Classic and Compendium of Materia Medica* as a traditional Chinese medicine. Several recent studies have reported the therapeutic effects of honeycombs on dental caries, toothache, and antimicrobials [5]. Felicioli et al. reported that Gram-positive and Gram-negative bacteria were affected by exposure to beeswax (a main component of honeycomb) [6].

*A. cerana* and *A. mellifera* are the two most economically valuable honeybee species used in apiculture [7]. *A. cerana* and *A. mellifera* have some differences in biological and physiological characteristics [8]. *A. mellifera* collect resins from buds, exudates, and other parts of plants, and mixes them with their salivary enzymes and beeswax, which creates propolis. Propolis is then used by bees to protect and reinforce their hives, repair their structure, and cover honeycombs as a building material and defensive substance [9]. Propolis showed strong antibacterial activity, and more than 600 bacterial strains were analyzed, mainly against Gram-positive bacteria [10]. Nora et al. reported that propolis could protect honeybees against associated viruses (titers of deformed wing virus and sacbrood virus), and enhance the colonies [11]. Mura et al. reported that propolis consumption reduces *Nosema ceranae* infection of *A. mellifera* [12]. *A. mellifera* colonies infected by Varroa increase the number of resin foragers [13,14]. If intruders are killed in the hive and are too large to be carried out, *A. mellifera* will use propolis to mummify it to prevent their decay. Propolis is responsible for the lower incidence of bacteria and molds within the hive than in the atmosphere outside. Therefore, propolis plays an important role in keeping the hive clean. It is noteworthy that *A. cerana* does not produce propolis [15]. Without propolis, there must be other antibacterial materials present in the hive of *A. cerana* that is use for maintaining eggs and larvae until they grow to imagoes, and protecting the hive against invasion by various external microorganisms. As far as we know, it has not been reported in the literature as to how *A. cerana* keep their hives clean. Thus, we predicted that there might be differences in the VOCs in honeycombs of *Apis cerana* and *A. mellifera*. These potential chemical markers in ACC may play an important role in keeping the honeycomb clean and protecting against invasion by various external microorganisms.

Solid-phase microextraction (SPME) is a simple, affordable, and versatile solvent-free technique that integrates sampling, extraction, and concentration of VOCs before gas chromatography-mass spectrometry (GC–MS) analysis [16]. HS-SPME/GC-MS has been widely used in food analysis. In the specific case of honeybee products, SPME has been successfully applied to the characterization and analysis of the VOCs from honey [7], propolis [17], and royal jelly [18].

In this study, an SPME technique combined with GC–MS and multivariate statistical analysis was applied to compare the chemical constituents of ACC and AMC. HS-SPME/GC-MS was used to analyze the chemical components of honeycomb from different bee species. Multivariate statistical methods, such as PCA, were performed to classify ACC and AMC. Furthermore, OPLS-DA was used to find the potential chemical markers that could be used for the classification of honeycombs from those two species. This research helps to understand the chemical composition of honeycomb, discover potential chemical markers in ACC, and distinguish the differences in chemical composition between ACC and AMC.

## 2. Materials and Methods

### 2.1. Honeycomb Samples

Five *A. cerana* honeycombs (ACC-1, ACC-2, ACC-3, ACC-4, and ACC-5) and five *A. mellifera* honeycombs (AMC-1, AMC-2, AMC-3, AMC-4 and AMC-5) were collected by beekeepers from different sub-regions of Jiangxi Province, China in 2019. Samples were cut from the surface of a honeycomb without touching the comb foundation, and honey, pollen, and bee larvae were utterly removed. They were stored at −20 °C until analysis.

### 2.2. Sample Preparation

A manual SPME holder (Thermo Fisher Scientific, Waltham, MA, USA) equipped with a 2 cm long SPME fiber composed of 50/30 μm divinylbenzene/carboxy/polydimethylsiloxane (DVB/CAR/PDMS) (Supelco Inc., Bellefonte, PA, USA). The SPME fiber was conditioned at the temperature suggested in the manufacturer’s instructions before analysis.

The frozen samples were finely powdered using a mortar and a pestle before the adsorption procedure. For each extraction, 0.50 g finely powdered honeycomb was placed in a 15 mL flat bottom headspace vial, and 200 µL 1-octanol solution (10 µg/mL) was added as the internal standard. The vials were sealed with a polypropylene cap and a polytetrafluoroethylene (PTFE)/silicon septum (Supelco Inc., Bellefonte, PA, USA). Each vial was equilibrated for 20 min at 60 °C. The SPME device was then manually inserted into the vials, and the fiber was exposed to the headspace for 60 min at the same temperature during the extraction time. After sampling, the SPME fiber was immediately desorbed into the GC injection port at 250 °C for 3 min.

### 2.3. GC-MS Conditions

Chromatographic analyses were carried out on a GC 7890B (Agilent Technologies, Waldbronn, Germany), coupled with a 7000C Network mass spectrometer (Agilent Technologies), equipped with a HP-5MS capillary column (length: 30 m; inner diameter: 0.25 mm; film thickness: 0.25 μm; Agilent Technologies). The volatiles were desorbed from the SPME fiber at 250 °C in the injection port. The running program was held at 45 °C for 2 min, increased to 180 °C at 3 °C/min, and finally increased to 250 °C at 10 °C/min. Transfer line and ion-source temperatures were 250 and 230 °C, respectively. Helium (purity > 99.999%) was used as the carrier gas, flowing at 1 mL/min, and injection was in a splitless mode. The mass spectrometer was operated in an electron ionization (EI) mode at 70 eV with a scan range from 30 to 500 m/z.

### 2.4. Qualitative and Quantitative Analysis

The volatile organic components (VOCs) were identified by comparing retention times and mass fragmentations with those in the mass spectral database of the NIST Library (NIST14, National Institute of Standards and Technology, Gaithersburg, MD, USA). The retention index (RI) of compounds was calculated from a series of n-alkane (C6–C40) (Sigma, St. Louis, MO, USA), which had the same GC-MS analysis program as that applied to samples. Calculated RI was then compared with those found in the literature and with that which was recorded in the NIST network database (https://webbook.nist.gov/chemistry/ (accessed on 14 July 2020)). The relative content of each VOC was quantified as 1-octanol equivalent (internal standard) by the GC peak area (Shi et al., 2020).

### 2.5. Statistical Analysis

All the data were the mean of three replicates. The mean values of volatile contents were used as input data in the unsupervised principal component analysis (PCA) and supervised orthogonal partial least-squares discriminant analysis (OPLS-DA). OPLS-DA model was evaluated in terms of the three parameters of R2X (variance explained by the X matrix), R2Y (variance explained by the Y matrix), and Q2 (goodness of predictive power). The variable importance in projection (VIP) scores > 1 were selected as potential markers (*p* < 0.05), as these variables are generally considered significant for group discrimination. MetaboAnalyst4.0 (http://www.metaboanalyst.ca (accessed on 15 October 2020)) and SPSS 20.0 software (SPSS Inc., Chicago, IL, USA) were used to perform statistical analyses.

## 3. Results

### 3.1. Characterisation of the Volatile Composition

**Figure 1:**
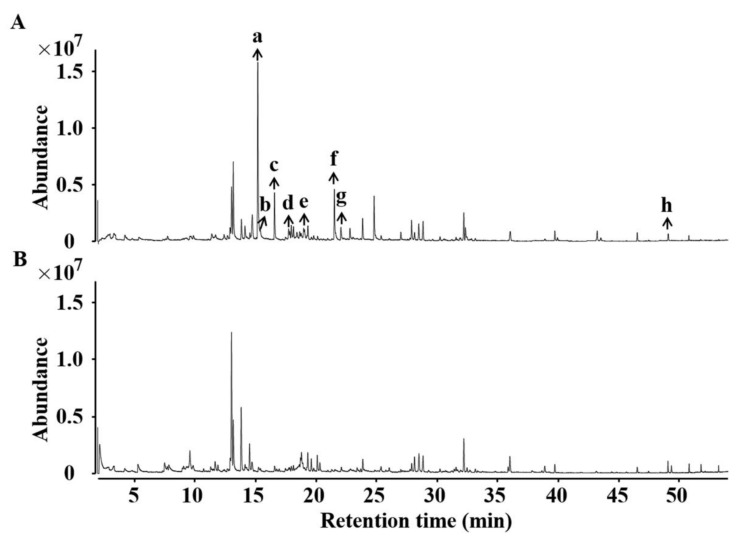
Typical chromatogram of the volatile components from ACC (**A**) and AMC (**B**) by SPME-GC–MS.

The total ion chromatogram (TIC) of ACC and AMC is shown in Figure 1. A total of 114 VOCs were found and identified in the honeycombs: 11 aldehydes, 13 hydrocarbons, 11 ketones, 26 esters, 38 terpenes, 3 alcohols, and 12 others (Table 1). The detailed information is shown in Table S1. In the different types of analyzed honeycomb, a lot of compounds were identified: including 97 in ACC and 84 in AMC. Among them, 13 compounds (benzeneacetaldehyde, nonanal, decanal, styrene, heptadecane, nonadecane, heptacosane, 2-nonanone, ethyl octoate, ethyl nonanoate, ethyl caprate, α-cedrene, 2-phenylethanol) were detected in all samples.

**Figure 2:**
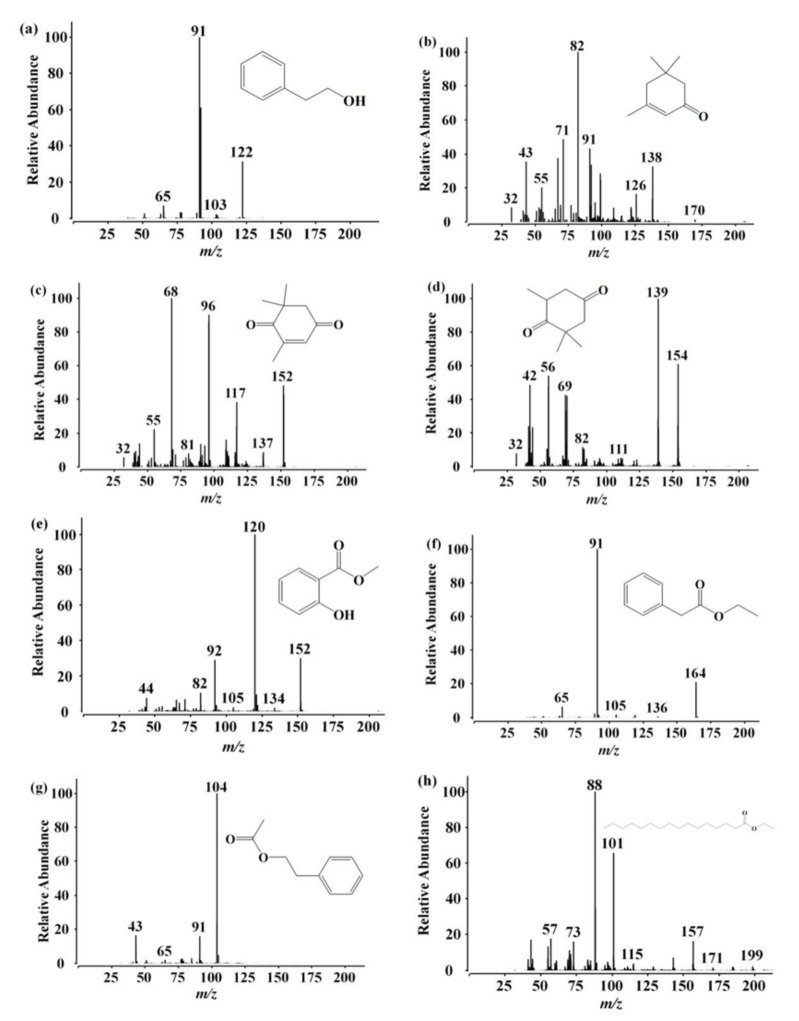
The mass fragments of the 8 potential markers in the ACC (identified in Figure 1A): (**a**) 2-phenylethanol; (**b**) isophorone; (**c**) 4-oxoisophorone; (**d**) dihydrooxophorone; (**e**) betula; (**f**) ethyl phenylacetate; (**g**) phenethyl acetate; (**h**) ethyl palmitate.

Quantitatively, the relative contents of each component differed among VOCs from different bee species’ honeycombs. For instance, phenethyl acetate was detected only in all ACC samples, whereas furfural and linalool were detected only in all AMC samples (Table S1). Consequently, in order to find marker compounds that could be used for the comparison of ACC and AMC, an independent samples *t*-test analysis was employed for analyzing the relative contents of the peaks of honeycomb [19]. As shown in Figure 2 and Table S1, there were significant differences in VOCs between them. The main VOCs of ACC included 2-Phenylethanol (average 5619.78 µg kg^−1^), trans-linalool oxide (average 4134.56 µg kg^−1^), and linalool oxide (average 3884.36 µg kg^−1^). The main components of AMC included octanal (average 2455.65 µg kg^−1^), linalool oxide (average 2387.28 µg kg^−1^), and furfural (average 1786.01 µg kg^−1^). In general, the contents of hydrocarbons, ketones, ester, terpenes, alcohols, and others, were higher in ACC than in AMC (Table 1). It is worth noting that there is a significant difference between ACC and AMC in the content of ketones, esters, and alcohols (*p* < 0.05) (Table 1). The average contents of ketone in ACC and AMC were 4257.70 µg kg^−1^ and 883.58 µg kg^−1^, respectively, including 2-heptanone, sulcatone, acetophenone, 2-nonanone, isophorone, 3-Nonen-2-one, 4-oxoisophorone, dihydrooxophorone, 2-undecanone, damascenone, β-ionone. The average contents of esters in ACC and AMC were 7707.52 µg kg^−1^ and 2154.96 µg kg^−1^, respectively, including ethyl octoate, ethyl nonanoate, ethyl caprate, ethyl phenylacetate, phenethyl acetate, etc. The average contents of alcohol in ACC and AMC were 6008.73 µg kg^−1^ and 2154.96 µg kg^−1^, respectively, including ethylhexanol, benzyl alcohol, 2-phenylethanol (Table S1). However, the total VOC contents ranged from 10,077.61 to 48,933.33 µg kg^−1^ of AMC, and 12,596.05 to 80,840.77 µg kg^−1^ of ACC. The total VOC amount showed no significant differences between the two different honeycomb extracts.

Terpenes were the main VOCs present in ACC, followed by esters (Table 1). The most abundant terpenes in ACC, of which the average content was above 1000 µg kg^−1^, were trans-linalool oxide (4134.56 µg kg^−1^), linalool oxide (3884.36 µg kg^−1^), cedrol (2347.24 µg kg^−1^), caryophyllene (1133.73 µg kg^−1^) and cedr-8-ene (1010.68 µg kg^−1^). Terpenes were also the main VOCs present in AMC, followed by aldehydes.

### 3.2. PCA

**Figure 3:**
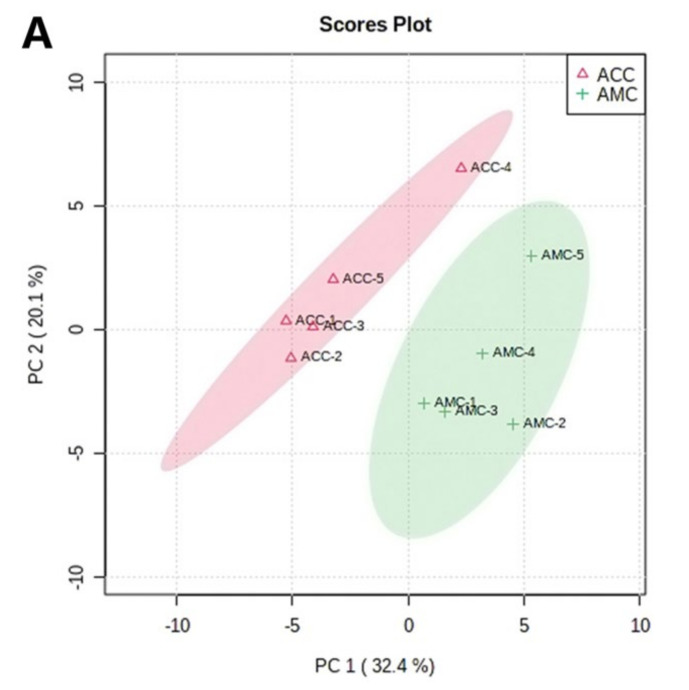

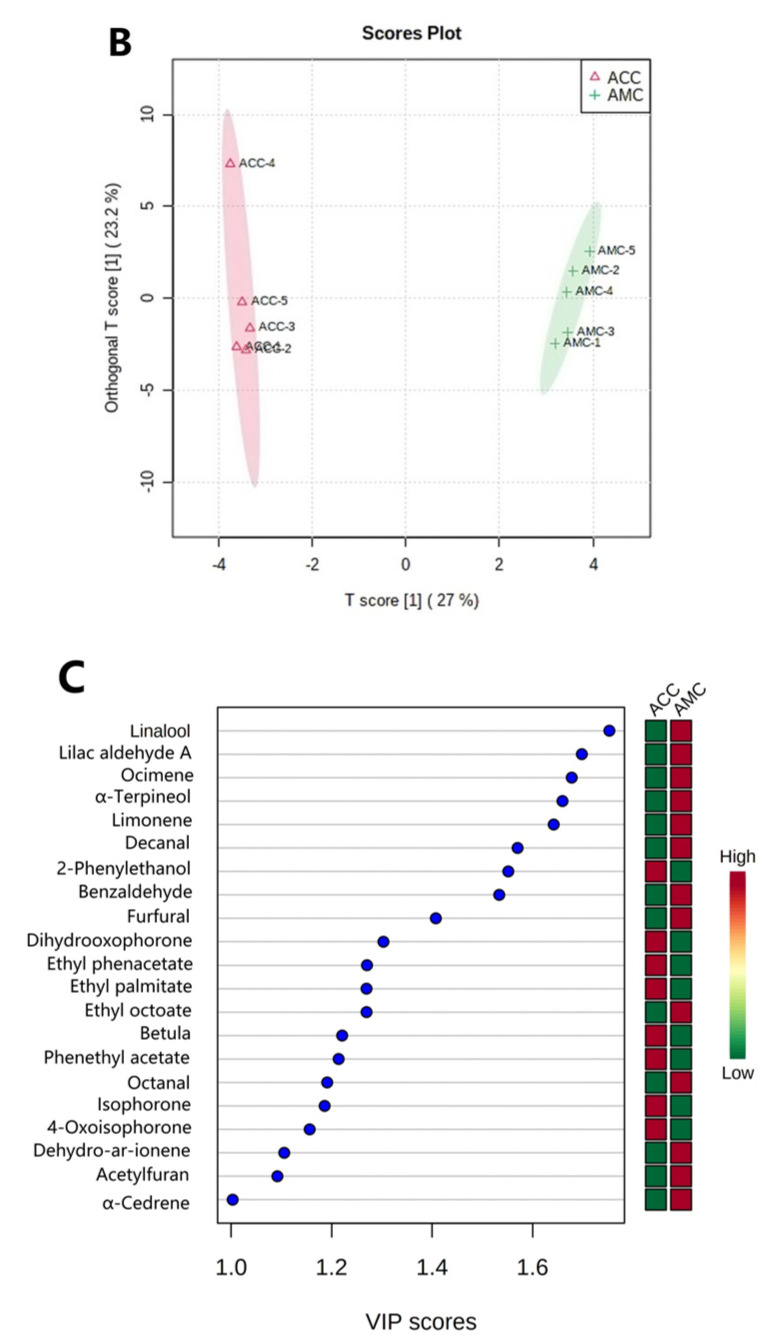
Discrimination of ACC and AMC. (**A**) PCA score plot of ACC and AMC; (**B**) OPLS-DA score plot of ACC and AMC; (**C**) VIP plot with VIP score > 1.

PCA was used to elucidate the difference between the two kinds of honeycomb in this study. Unsupervised PCA provides an overview of trends of data sets and reduces the dimension of multivariate data while keeping most of the variance within it. A total of 114 VOCs were used in the PCA model with two major principal components (PC1/PC2) accounting for 32.4% and 20.1% of total variability, respectively, which represented 52.5% of the total variance (Figure 3A). There was a clear separation trend between ACC (green cluster) and AMC (red cluster) samples. The PCA plot showed all clusters in the dataset with ACC and AMC samples falling on the 95% confidence ellipse regions based on the data points for individual groups. Therefore, the VOCs in ACC were different from those in AMC.

### 3.3. OPLS-DA

The statistics were then supervised by an OPLS-DA analysis (Figure 3B,C). OPLS-DA results showed that the two classes of honeycomb were well-separated, with Q2 = 0.896, R2Y = 0.926, and R2X = 0.251, and with ACC and AMC samples falling in the 95% confidence ellipse regions (Figure 3B). A total of 21 compounds with a VIP value greater than 1 were selected as potential markers (*p* < 0.05).

In summary, honeycomb from different entomological origins were discriminated using a chemometric method based on GC-MS data. Eight VOCs (2-phenylethanol, dihydrooxophorone, ethyl phenylacetate, ethyl palmitate, betula, phenethyl acetate, isophorone, and 4-Oxoisophorone) were selected as potential markers for ACC, whereas thirteen VOCs (linalool, lilac aldehyde A, ocimene, α-terpineol, limonene, decanal, benzaldehyde, furfural, ethyl octoate, octanal, dehydro-ar-ionene, acetyl furan, and α-cedrene) were selected as potential markers for AMC (Figure 3C).

## 4. Discussion

Comparatively to our result, Felicioli et al. [6] reported that aldehydes were the main class of VOC in the *A. mellifera* beeswax ethanol extracts. Kaskoniene et al. [20] had reported that aldehydes were an important source of honey flavor and provided the fragrant smell of flowers and fruits. In our study, terpenes were the main VOCs present in AMC, followed by aldehydes. This is similar to the volatile characteristics of *A. mellifera* beeswax, particularly linear aldehydes, such as octanal, nonanal, and decanal, because beeswax is recovered from the honeycomb [6]. Terpenes were also the main VOCs present in ACC, followed by esters. According to Kumar et al. [21], terpenes are generally considered to be active constituents in natural products that exhibit antibacterial, antitumor, anti-wrinkle, antioxidative, anti-tussive, analgesia, and immune-regulatory effects.

The 12 potential markers in AMC are shown in Table 2. Linalool, α-terpineol, limonene, decanal, benzaldehyde, octanal, and α-cedrene isolated from propolis [22,23,24,25], another bee product closely related with honeycomb, showed antibacterial activity against a broad bacteria spectrum. Indeed, linalool and α-terpineol showed remarkable antibacterial effects against periodontopathic and cariogenic bacteria [26]. Limonene showed strong antibacterial and antifungal effects on foodborne pathogens [27]. Benzaldehyde was found in higher concentrations in *A. mellifera* honey [7]. It may be that some substances in the propolis of a honeycomb would enter the honey. Linear aldehydes, such as octanal and decanal, also showed high contents in *A. mellifera* beeswax, with octanal having the highest content [6]. Octanal isolated from other natural products showed antibacterial activity against *Escherichia coli* [28], *Staphylococcus aureus* [29], and Listeria monocytogenes [30]. Decanal isolated from other natural compounds showed better antibacterial activity to *E. coli, P. aeruginosa*, and *S. aureus* [22,31]. Furfural was only detected in AMC samples, another difference between *A. cerana* and *A. mellifera* honey. However, heat treatment, which was essential in this experiment, will produce the Maillard reaction leading to the production of furfural, thus it was not considered a suitable marker of honeycombs from either *A. mellifera* or *A. cerana.* Our results showed that *A. mellifera* produces propolis for the hive, which can enhance the antibacterial effect of the honeycomb.

**Figure 4:**
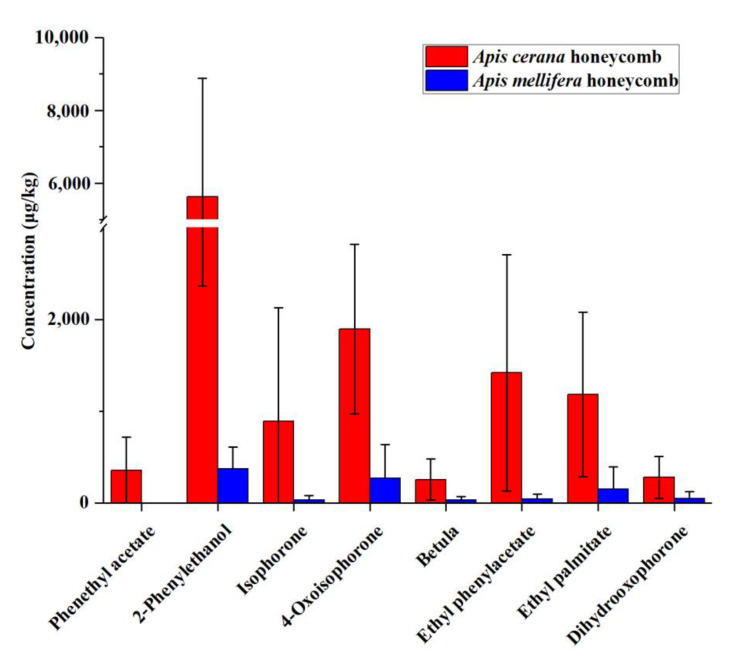
Quantitative results of the potential markers in ACC.

*A. cerana* does not produce propolis, the 8 potential markers in the ACC have been shown in Table 3, Figure 2 and Figure 4. As reported, the content of 2-phenylethanol in ACC was significantly higher than that in AMC (*p* < 0.05), over 15 times, in fact. 2-phenylethanol is a colorless liquid with a sweet rose-like floral fragrance. It occurs widely in nature and is found in a variety of essential oils, including rose, carnation, hyacinth, Aleppo pine. 2-phenylethanol is also one of the main VOCs in many kinds of monofloral honey [32,33]. It is used as an additive in cigarettes and a preservative in soaps. As a mixed-type inhibitor, 2-phenylethanol had been shown to be inhibitory to Gram-negative [34] and Gram-positive [35] bacteria. Zhu et al. [36] reported that 2-phenylethanol could lead to reversible inhibition of the enzyme. Berrah and Konetzka (1962a) reported that 2-phenylethanol blocked deoxyribonucleic acid synthesis. Phenethyl acetate was only detected in ACC samples, and the content of phenethyl acetate in *A. cerana* honey was significantly higher than that of *A. mellifera* honey [7]. This compound was observed in most honey samples but at different concentrations, as the samples originated from different floral and geographical origins. Phenethyl acetate and 2-phenylethanol were found to be highly effective against all the test fungi with MIC values of 270 to 1704 ppm, and thus potentially holds promise in plant disease management and plant quarantine [37]. Ethyl palmitate is known to be a component of honeybee brood pheromones. Isophorone and 4-Oxoisophorone (ketoisophorone) also revealed a higher response in ACC, and have been reported to be present in plant essential oils and in most honey [35,38]. Isophorone can be oxidized to 4-Oxoisophorone during the drying process [38]. Isophorones and their derivatives showed wide antimicrobial activity against different indicator pathogenic microorganisms, such as *E. coli*, *Bacillus subtilis*, *Pseudomonas aeruginosa*, and *Salmonella typhimurium* [39].

## 5. Conclusions

This study is the first attempt to compare the VOCs in ACC and AMC. HS-SPME/GC-MS technique was applied to study the differences in the volatility of honeycombs from ACC and AMC. A total of 114 common volatile substances were identified in ACC and AMC. Results of PCA and OPLS-DA have shown that there was a significant difference in VOCs between ACC and AMC honeycombs. Potential markers in AMC are mainly derived from propolis, such as benzaldehyde, octanal, limonene, ocimene, linalool, α-terpineol and decanal. 2-phenylethanol, phenethyl acetate, isophorone, 4-oxoisophorone, betula, ethyl phenylacetate, ethyl palmitate, and dihydrooxophorone were richer in ACC than AMC, which may be the reason why *A. cerana* hives are cleaner. This study provides a practical method to find antibacterial substances in the honeycomb. The comparison of VOCs will help to further understand their role in maintaining the cleanliness of the honeycomb and provide basic data support for the development of honeycomb as a new functional food resource.

## Figures and Tables

**Table 1:** Compound type of ACC and AMC detected by SPME-GC-MS.

Compound Type	Numbers	Total Content µg/kg (Range)	
		ACC (*n* = 5)	AMC (*n* = 5)
Aldehydes	11	4761.29 (1292.83–10,141.32)	7056.93 (3681.69–10,560.34)
Hydrocarbons	13	2914.96 (855.18–4979.14)	2436.3 (888.12–4019.27)
Ketones	11	4534.38 (2420.21–7892.92) ^a^	931.61 (238.2–1556.39) ^b^
Esters	26	7707.52 (2721.26–13,400.18) ^a^	2154.96 (268.96–5424.89) ^b^
Terpenes	38	16,081.19 (1706.25–41,958.44)	8510.35 (1848.85–24,193.50)
Alcohols	3	6008.73 (3127.71–11,434.83) ^a^	435.41 (189.43–780.95) ^b^
Others	12	990.67 (0–1701.63)	1834.5 (114.53–4954.57)

Notes: ^a^ and ^b^ indicate significant differences (*p* < 0.05).

**Table 2:** Potential markers in AMC identified by GC−MS.

Compounds	CAS No.	RT	RIa	RIb	Major Ions	Propolis	Reference
Acetylfuran	1192-62-7	5.299	916	915	95, 101, 42, 67	−	
Benzaldehyde	100-52-7	7.484	961	961	106, 105, 77	+	[22,24]
Octanal	124-13-0	9.606	1005	1001	43, 56, 84, 69	+	[22]
Limonene	138-86-3	10.725	1027	1030	68, 93, 44, 136	+	[22]
Ocimene	13877-91-3	11.894	1051	1044	93, 91, 97, 136	−	
Linalool	78-70-6	14.510	1103	1104	93, 71, 121, 43	+	[22]
Lilac aldehyde A	53447-46-4	16.954	1153	1154	71, 55, 93, 43	−	
α-terpineol	10482-56-1	18.801	1191	1187	121, 93, 59, 136	+	[22,24]
Ethyl octoate	106-32-1	19.315	1202	1196	88, 101, 57, 127	−	
Decanal	112-31-2	19.591	1208	1195	57, 82, 70, 43	+	[22]
Dehydro-ar-ionene	30364-38-6	26.043	1351	1349	157, 142, 172, 44	−	
α-cedrene	469-61-4	28.488	1408	1408	119, 93, 161, 105	+	[24]

Notes: RIa: retention index calculated as determined on HP-5MS column using the homologous series of n-alkanes (C6–C40); RIb: retention index from the NIST network database; +: Main constituents in propolis and is often detected in propolis; −: Not the main constituents in propolis.

**Table 3:** Potential markers in ACC identified by GC−MS.

Compounds	CAS No.	RT	RI ^a^	RI ^b^	Major Ions	Bioactivity	Reference
2-Phenylethanol	60-12-8	15.232	1118	1114	91, 92, 122, 65	Antibacterial, antifungal	[36,37]
Isophorone	78-59-1	15.391	1121	1118	82, 71, 91, 138	Antibacterial	[39]
4-Oxoisophorone	1125-21-9	16.573	1145	1142	68, 96,152,117	NT	
Dihydrooxophorone	20547-99-3	17.753	1169	1170	139, 154, 56, 42	NT	
Betula	119-36-8	19.063	1196	1187	120, 152,92, 65	Antibacterial	[39]
Ethyl phenylacetate	101-97-3	21.551	1250	1244	91,164, 65	NT	
phenethyl acetate	103-45-7	22.060	1261	1244	104, 43,91	Antifungal	[37]
Ethyl palmitate	628-97-7	49.060	1998	1996	88, 101, 43,57	NT	

Notes: RIa: retention index calculated as determined on HP-5MS column using the homologous series of n-alkanes (C6–C40); RIb: retention index from the NIST network database; NT: not tested.

## Data Availability

Data is contained within the article or Supplementary Materials.

## Supplementary Materials

The following supporting information can be downloaded at: https://www.mdpi.com/article/10.3390/foods11203204/s1, Table S1: Compounds in ACC and AMC detected by SPME-GC-MS (*n* = 3, the equivalent of 1-octanol). All GC-MS raw data can be found on https://data.mendeley.com/datasets/ny3pjs9z9g (accessed on 4 September 2022).
