# Supplementary material for: Honeycomb, a New Food Resource with Health Care Functions: The Difference of Volatile Compounds found in Apis cerana and A. mellifera Honeycombs
Source: Foods. 2022 Oct 14;11(20):3204. doi: 10.3390/foods11203204 (PMC9601661; doi:10.3390/foods11203204)
Supplement: Supplementary file 1 [file foods-11-03204-s001.zip › foods-1882411-supplementary.pdf]

Honeycomb, a new food resource with health care function: the difference of volatile compounds between honeycombs from *Apis cerana* and *A. mellifera*

Xiali Guo 1, Yanlang Lian 1, Shengxiang Yi 1, Shengrong Qiu 1, Mingyan Liu 1, Fangjian Ning 2*, Liping Luo 1,3*

[1] School of Life Sciences, Nanchang University, Nanchang, 330031, China
2 School of Food and Health, Beijing Technology and Business University, Beijing 100048, China

[3] State Key Laboratory of Food Science and Technology, Nanchang University, Nanchang, 330031, China

* Corresponding author:

1-Liping Luo:

lluo2@126.com
Tel: +86-0791-83969519; Fax: +86-0791-83969519
2-Fangjian Ning

ningfj@btbu.edu.cn
Tel: +86-010-68984003; Fax: +86-010-68984003

Supplementary Table S1. Compounds in ACC and AMC detected by SPME-GC-MS (n = 3, the equivalent of 1-octanol).

Code	CAS No	Compounds	RT	RI[a]	RI[b]	Content [μg/kg (range)]		*p* Value
						AMC=5	ACC=5	
Aldehydes								
1	498-60-2	Furfural	2.12	815	832	1786.01 (37.19- 4675.71)	nd	0.011
2	111-71-7	Heptanal	4.83	906	901	nd	80.23 (0- 146.44)	0.072
3	100-52-7	Benzaldehyde	7.48	961	961	406.44 (39.75- 566.20)	82.68 (0- 256.28)	0.075
4	620-02-0	5-Methylfurfural	7.83	969	966	117.68 (0- 269.67)	nd	0.066
5	124-13-0	Octanal	9.61	1005	1001	2455.65 (1580.44- 3980.78)	1137.23 (0- 5686.13)	0.043
6	122-78-1	Benzeneacetaldehyde	11.65	1046	1049	509.97 (107.77- 893.87)	783.86 (182.24- 1700.34)	0.470
7	124-19-6	Nonanal	14.71	1107	1102	824.11(469.89-1188.21)	2218.04 (884.86-4168.88)	0.020
8	53447-46-4	Lilac aldehyde A	16.95	1153	1154	131.74 (90.92-189.89)	25.49 (0-101.26)	0.006
9	112-31-2	Decanal	19.59	1208	1195	820.38 (295.15-1528.94)	263.97 (53.08-363.35)	0.014
10	3913-81-3	trans-2-Decenal	22.19	1264	1265	nd	37.16 (0-185.79)	0.374
11	112-44-7	Undecanal	24.23	1309	1308	4.94 (0-24.71)	132.63(0-259.79)	0.082
Hydrocarbons								
1	100-42-5	Styrene	4.22	880	890	274.22 (198.39-326.78)	560.05 (129.64-917.85)	0.233
2	124-18-5	Decane	9.37	1001	1000	nd	50.62 (0-253.11)	0.374
3	1120-21-4	Undecane	14.41	1101	1100	8.57 (0-42.83)	20.06 (0-100.31)	0.374
4	112-40-3	Dodecane	19.25	1200	1200	99.06 (0-224.11)	126.56 (0-397.62)	0.618
5	13360-61-7	1-Pentadecene	31.44	1480	1489	nd	11.29 (0-56.46)	0.374
6	629-62-9	Pentadecane	32.21	1499	1500	1135.15 (187.42-2395.90)	1334.49 (0-3282.68)	0.704

7	629-78-7	Heptadecane	39.72	1698	1700	330.25 (94.3-622.81)	203.86 (49.75-376.72)	0.469
8	593-45-3	Octadecane	43.19	1799	1800	110.07 (0-550.36)	141.35 (0-445.69)	0.703
9	638-36-8	Phytan	43.50	1808	1809	nd	28.76 (0-143.78)	0.374
10	629-92-5	Nonadecane	46.51	1900	1900	212.69 (47.5-472.63)	181.75 (47.38-342.78)	0.999
11	112-95-8	Eicosane	49.10	2000	2000	45.17 (0-225.85)	nd	0.374
12	629-94-7	Heneicosane	50.80	2100	2100	132.75 (32.82-306.27)	128.41 (0-320.84)	0.927
13	593-49-7	Heptacosane	53.24	2700	2700	88.37 (35.24-166.95)	127.77 (45.76-236.34)	0.446
Ketones								
1	110-43-0	2-Heptanone	4.41	892	894	nd	136.25 (0-533.48)	0.195
2	110-93-0	Sulcatone	9.01	993	987	99.29 (0-227.13)	35.03 (0-175.13)	0.228
3	98-86-2	Acetophenone	12.83	1069	1065	48.21 (0-141.19)	8.81 (0-44.04)	0.220
4	821-55-6	2-Nonanone	14.15	1096	1090	396.88 (155.94-1046.83)	1113.45 (630.42-2454.69)	0.071
5	78-59-1	Isophorone	15.39	1121	1118	31.28 (0-110.10)	847.59 (0-3093.12)	0.039
6	14309-57-0	3-Nonen-2-one	16.52	1144	1136	nd	50.08 (0-250.42)	0.374
7	1125-21-9	4-Oxoisophorone	16.57	1145	1142	271.59 (0-843.45)	1707.45 (90.16-2896.75)	0.047
8	20547-99-3	Dihydrooxophorone	17.75	1169	1170	48.03 (0-163.34)	276.68 (0-509.03)	0.047
9	112-12-9	2-Undecanone	23.67	1296	1294	nd	38.84 (0-165.77)	0.307
10	23726-93-4	Damascenone	27.57	1386	1384	nd	320.20 (0-1447.59)	0.174
11	14901-07-6	β-Ionone	31.68	1486	1488	36.33 (0-181.65)	nd	0.374
Ester								
1	108-29-2	4-Pentanolide	7.20	956	950	6.14 (0-30.71)	16.28 (0-81.42)	0.545
2	123-66-0	Ethyl caproate	9.60	1005	996	nd	556.20 (0-2568.52)	0.211
3	1073-11-6	Lavender lactone	11.52	1043	1041	19.95 (0-99.76)	nd	0.374

4	93-58-3	Methyl benzoate	14.27	1098	1091	49.58 (0-128.00)	26.31 (0-131.54)	0.403
5	106-30-9	Ethyl enanthate	14.54	1104	1095	nd	264.45 (0-518.47)	0.027
6	111-11-5	Methyl caprylate	15.81	1130	1126	21.38 (0-106.90)	nd	0.374
7	93-89-0	Benzoic ether	17.97	1174	1170	153.38 (0-346.86)	446.42 (0-855.68)	0.037
8	101-41-7	Methyl phenylacetate	18.43	1183	1179	nd	103.39 (0-413.54)	0.156
9	123-25-1	Ethyl succinate	18.75	1190	1181	64.58 (0-322.90)	119.22 (0-265.70)	0.499
10	119-36-8	Betula	19.06	1196	1187	27.95 (0-79.15)	254.76 (0-583.99)	0.025
11	106-32-1	Ethyl octoate	19.32	1202	1196	570.63 (107.67-1253.65)	486.81 (120.5-823.36)	0.705
12	1731-84-6	Methyl nonanoate	20.60	1230	1224	4.66 (0-23.3)	4.79 (0-23.94)	0.374
13	115-95-7	Linalyl acetate	21.29	1245	1248	nd	139.63 (0-698.15)	0.374
14	101-97-3	Ethyl phenacetate	21.55	1250	1244	44.41 (0-123.39)	1417.72 (92.14-3548.17)	0.003
15	103-45-7	Benzylcarbinyl acetate	22.06	1261	1256	nd	356.74 (49.5-964.47)	0.009
16	123-29-5	Ethyl nonanoate	23.84	1300	1294	376.77 (54.2-852.7)	1011.57 (99.49-1948.88)	0.008
17	110-42-9	Methyl caprate	25.07	1328	1325	11.52 (0-57.62)	nd	0.374
18	110-38-3	Ethyl caprate	28.13	1399	1397	415.50 (76.37-1146.75)	462.56 (164.36-756.92)	0.332
19	51154-96-2	Massoilactone	31.46	1480	1483	nd	142.28 (0-711.41)	0.374
20	17092-92-1	Ledene	33.33	1528	1525	27.38 (0-97.5)	nd	0.225
21	124-06-1	Ethyl myristate	43.13	1797	1793	24.18 (0-120.92)	197.86 (0-410.93)	0.242
22	84-69-5	Diisobutyl phthalate	45.63	1873	1872	54.20 (0-223.92)	235.01 (0-960.05)	0.075
23	112-39-0	Methyl palmitate	47.44	1936	1928	28.68 (0-98.20)	33.99 (0-131.25)	0.962
24	84-74-2	Dibutyl phthalate	48.41	1973	1968	51.44 (0-225.12)	219.98 (0-814.72)	0.033
25	628-97-7	Ethyl palmitate	49.06	1998	1996	147.22 (0-586.24)	1184.05 (254.52-2434.95)	0.035

26	111-62-6	Ethyl Oleate	51.77		2179	55.39 (0-245.45)	27.50 (0-137.49)	0.773
Terpenes								
1	80-56-8	2-Pinene	6.00	931	939	nd	12.42 (0-62.10)	0.374
2	3391-86-4	Matsutake alcohol	8.61	985	986	nd	35.49 (0-177.43)	0.374
3	123-35-3	Myrcene	9.04	994	992	17.55 (0-87.74)	nd	0.374
4	138-86-3	Limonene	10.73	1027	1030	163.72 (91.67-232.19)	23.62 (0-66.79)	0.009
5	13877-91-3	Ocimene	11.89	1051	1044	223.24 (77.01-339.63)	4.97 (0-24.83)	0.001
6	5989-33-3	Linalool oxide	13.03	1073	1065	2387.28 (0-10463.23)	3884.36 (79.88-3842.28)	0.361
7	34995-77-2	trans-Linalol oxide	13.84	1089	1087	1049.32 (0-4471.13)	4134.56 (76.68-17541.38)	0.374
8	78-70-6	Linalool	14.51	1103	1104	1044.88 (277.29-1987.13)	nd	0.001
9	39028-58-5	trans-linalool oxide (pyranoid)	18.15	1178	1183	129.64 (0-286.06)	457.45 (0-1185.90)	0.324
10	1197-01-9	p-cymenol	18.65	1188	1188	12.71(0-63.55)	67.57 (0-337.86)	0.631
11	10482-56-1	Alpha terpineol	18.80	1191	1187	665.98 (290.19-1156.36)	141.72 (0-708.61)	0.046
12	106-26-3	cis-Citral	21.32	1245	1242	nd	23.78 (0-118.89)	0.374
13	17699-14-8	Cubebene	25.93	1348	1349	9.27 (0-46.36)	nd	0.374
14	3856-25-5	Copaene	27.02	1373	1376	53.39 (0-173.20)	456.79 (0-1014.44)	0.119
15	94482-89-0	Isoitalicene	27.34	1381	1385	20.30 (0-101.48)	67.59 (0-308.71)	0.696
16	515-13-9	beta elemene	27.77	1390	1394	85.31 (0-284.24)	74.11 (0-267.10)	0.959
17	118-65-0	Isocaryophyllene	28.32	1404	1404	nd	9.32 (0-46.62)	0.374
18	469-61-4	Cedr-8-ene	28.49	1408	1408	1105.61 (521.65-2364.14)	1010.68 (158.19-1904.82)	0.745
19	546-28-1	Cedr-8(15)-ene	28.81	1415	1413	310.91 (0-1075.51)	491.51 (0-2226.64)	0.747
20	87-44-5	Caryophyllene	28.82	1416	1417	348.32 (0-1323.38)	1133.73 (0-2192.97)	0.236

21	470-40-6	Thujopsene	29.28	1427	1426	81.26 (32.12-157.07)	78.79 (0-326.20)	0.619
22	489-39-4	Aromandendrene	29.61	1435	1440	nd	48.46 (0-154.12)	0.185
23	6753-98-6	Humulene	30.24	1450	1452	55.88 (0-208.03)	208.10 (0-470.81)	0.166
24	28973-97-9	cis-β-Farnesene	30.57	1459	1457	nd	52.93 (0-104.59)	0.073
25	24048-44-0	Acoradien	30.80	1464	1459	nd	22.94 (0-114.70)	0.374
26	515-17-3	γ-Selinene	31.19	1474	1470	nd	98.88 (0-494.39)	0.374
27	30021-74-0	γ-Muurolene	31.25	1475	1474	36.47 (0-118.37)	30.70 (0-128.89)	0.691
28	22567-17-5	γ-Gurjunene	31.57	1483	1479	nd	464.30 (0-1838.47)	0.096
29	10219-75-7	Eremophilene	31.67	1485	1486	nd	74.44 (0-372.19)	0.374
30	997297.00	Valencen	31.90	1491	1491	nd	23.89 (0-119.47)	0.374
31	18431-82-8	Chamigren	31.94	1492	1493	49.07 (0-137.11)	nd	0.179
32	10208-80-7	Muurolene	32.22	1499	1504	nd	184.62 (0-923.12)	0.374
33	16982-00-6	Cuparene	32.39	1503	1502	7.71 (0-38.56)	37.02 (0-185.11)	0.546
34	39029-41-9	γ-Cadinene	32.72	1512	1511	38.35 (0-143.11)	43.80 (0-148.39)	0.794
35	483-76-1	Cadina-1(10),4-diene	33.14	1523	1519	117.79 (0-264.12)	317.66 (32.77-708.76)	0.166
36	21391-99-1	(E)-Calacorene	33.89	1542	1542	nd	17.75 (0-47.65)	0.181
37	40716-66-3	trans-Nerolidol	34.81	1566	1562	28.23 (0-91.79)	nd	0.198
38	77-53-2	Cedrol	36.02	1597	1596	468.16 (0-1342.33)	2347.24 (0-5927.99)	0.199
Alcohols								
1	104-76-7	Ethylhexanol	11.07	1034	1035	7.54 (0-37.68)	50.23 (0-128.85)	0.147
2	100-51-6	Benzyl alcohol	11.38	1040	1033	59.20 (0-296.00)	338.71 (194.69-414.76)	0.029
3	60-12-8	2-Phenylethanol	15.23	1118	1114	368.67 (189.43-780.95)	5619.78 (2885.03-10955.25)	0.001
Others								

1	116-53-0	Ethylmethylacetic acid	3.37	824	832	22.91 (0-114.53)	nd	0.374
2	1192-62-7	Acetylfuran	5.30	916	915	339.38 (0-933.86)	19.62（0-98.10）	0.038
3	3658-80-8	Dimethyl trisulfide	7.72	966	972	364.32 (0-1077.54)	230.91 (0-485.32)	0.686
4	108-95-2	Phenol	9.08	994	994	64.44 (0-209.06)	nd	0.185
5	541-73-1	Metadichlorobenzene	9.86	1010	1007	176.70 (0-363.21)	308.63 (0-860.75)	0.405
6	1072-83-9	2-Acetylpyrrole	12.92	1071	1065	452.19 (0-1504.82)	306.28 (0-817.03)	0.328
7	124-07-2	Octanoic acid	18.91	1193	1180	54.54 (0-204.57)	nd	0.206
8	5756-24-1	Dimethyl tetrasulfide	19.80	1212	1206	233.19 (0-798.60)	53.71 (0-204.19)	0.359
9	2785-89-9	p-Ethylguaiacol	23.05	1283	1280	nd	12.71 (0-63.55)	0.374
10	112-05-0	Nonanoic acid	23.06	1283	1280	nd	21.89 (0-109.45)	0.374
11	30364-38-6	Dehydro-ar-ionene	26.04	1351	1349	111.47 (0-256.72)	36.90 (0-78.53)	0.174
12	334-48-5	Capric acid	27.19	1377	1380	15.36 (0-76.80)	nd	0.374

Notes: RT— Retention time (min); RI^a—retention index calculated as determined on HP-5MS column using the homologous series of n-alkanes (C6−C40); RI^b—retention index from the NIST network database; nd—not detected; *p* Value —two-sample t-test significant values at a level of 0.05.
